# Variability in Exposure of Epitope G40-R43 of Domain I in Commercial Anti-Beta2-Glycoprotein I IgG ELISAs

**DOI:** 10.1371/journal.pone.0071402

**Published:** 2013-08-12

**Authors:** Leonie Pelkmans, Hilde Kelchtermans, Philip G. de Groot, Stephane Zuily, Veronique Regnault, Denis Wahl, Vittorio Pengo, Bas de Laat

**Affiliations:** 1 Cardiovascular Research Institute Maastricht, Maastricht University Medical Centre, Maastricht, The Netherlands; 2 Synapse BV, Maastricht, The Netherlands; 3 Department of Clinical Chemistry and Haematology, University Medical Centre Utrecht, Utrecht, The Netherlands; 4 Vascular Medicine Division, Regional Competence Centre for Rare Vascular Diseases, CHU de Nancy, Nancy, France; 5 INSERM U1116, Université de Lorraine, Nancy, France; 6 Clinical Cardiology, Department of Cardiothoracic and Vascular Sciences, Thrombosis Centre, University of Padua, Padua, Italy; 7 Sanquin Research, Amsterdam, The Netherlands; Institut National de la Santé et de la Recherche Médicale, France

## Abstract

**Background:**

A major problem for diagnosing the antiphospholipid syndrome (APS) is the high variability between commercial anti-β_2_glycoprotein I (β_2_GPI) assays. Predominantly antibodies reactive against cryptic epitope Glycine40-Arginine43 (G40-R43) in domain I are associated with an increased risk for thrombosis. Upon interaction with anionic surfaces β_2_GPI opens up, thereby exposing G40-R43.

**Objectives:**

To examine whether suboptimal exposure of epitope G40-R43 explains the variations in results observed between commercial assays.

**Methods:**

Two patient-derived monoclonal antibodies were tested on neutral versus anionic plates. Antibody P1-117 reacts with G40-R43 in the open conformation while P2-6 recognizes β_2_GPI irrespective of its conformation. These antibodies were tested in commercial anti-β_2_GPI assays (A–E).

**Results:**

In assay A, both antibodies showed equal reactivity towards β_2_GPI, indicating that all the β_2_GPI exposes G40-R43. In other assays P1-117 displayed lower reactivity than P2-6, demonstrating reduced G40-R43 availability. To exclude influences of other assay features, reactivity was re-examined on plates of assay A and B using the protocol/reagents from each assay. In all combinations, reactivity of both antibodies on a plate was comparable to results obtained with its own protocol/reagents, suggesting that the coating, rather than other assay components, accounts for the observed differences. In two patient cohorts we demonstrated that a number of domain I-reactive samples are missed in assays characterized by a decreased exposure of epitope G40-R43.

**Conclusions:**

Exposure of epitope G40-R43 on β_2_GPI is highly variable between commercial anti-β_2_GPI assays. As a consequence, patients can be falsely assigned negative in assays characterized by a reduced exposure of G40-R43.

## Introduction

The antiphospholipid syndrome (APS) is defined by the simultaneous presence of vascular thrombosis or pregnancy morbidity and the detection of antiphospholipid antibodies in plasma [1]. Diagnosing a patient with APS heavily depends on the detection of antiphospholipid antibodies. As a consequence, the quality of the assays used to detect these antibodies is of utmost importance [2]. Three different types of assays to detect antiphospholipid antibodies are included in the official revised Sidney criteria; (1) phospholipid-dependent prolongation of coagulation (Lupus anticoagulant, LAC); (2) detection of IgM/IgG anti-β_2_glycoprotein I (β_2_GPI) antibodies; (3) detection of IgM/IgG anti-cardiolipin antibodies [1].

To reduce the false positivity and hence increase the specificity of these assays, several studies have been performed. Antibodies correlating with an increased risk for thrombosis have been shown to especially recognize one epitope on domain I of β_2_GPI, more precisely epitope Glycine40-Arginine43 (G40-R43) [3], [4], [5]. By detecting only these “anti-domain I” antibodies the false positivity rate reduces, resulting in an increased specificity. Moreover, in single center and multicenter studies it was demonstrated that detection of anti-domain I antibodies increased the association with thrombosis up to 4 times compared to detecting anti-β_2_GPI antibodies regardless of their specificity [3], [6], [7]. β_2_GPI has been shown to exist in different conformations, a circular, an S-shaped and J-shaped conformation [8], [9], [10], [11]. It is thought that β_2_GPI in plasma is present in its ‘closed’ native conformation, either S-shaped or circular, and that interaction with anionic phospholipids results in an activated open J-shaped conformation [8], [9]. We have published that such a conformational change is mandatory for the exposure of epitope G40-R43 on domain I and hence to enable anti-β_2_GPI antibodies to interact with the epitope [7]. Indeed, in its native conformations this epitope is hidden; owing to the twist between domains II and III in the S-shaped conformation, the carbohydrate residues are positioned in such a way that they shield the epitope for autoantibodies. In the circular conformation, interaction of domain I with domain V prevents exposure of the domain I epitope G40-R43.

A major problem within APS is the variability between different commercially available assays [2], [12], [13], [14]. In other words, a sample assigned positive in one test does not automatically test positive in the same type of assay from a different manufacturer. Since antibodies correlating with an increased risk for thrombosis recognize specifically the cryptic epitope G40-R43 on domain I of β_2_GPI, we hypothesized that the discrepancies between manufacturers of anti-β_2_GPI IgG assays result from differences in exposure of this epitope on β_2_GPI. We recently constructed human-derived monoclonal antibodies able to discriminate between both conformations of β_2_GPI characterized by an exposed or hidden epitope G40-R43 [15]. With these antibodies, we were able to establish differences in exposure of epitope G40-R43 on β_2_GPI in commercial anti-β_2_GPI IgG assays. More importantly, by testing several patient samples, we have demonstrated that these differences in exposure possibly affect the diagnosis of APS.

## Materials and Methods

### Antibodies

Two recently described monoclonal human-derived antibodies, P1-117 and P2-6, were used to study the exposure of epitope G40-R43 on domain I [15]. In short we selected two patients with anti-β_2_GPI antibodies in their plasma and a history of thrombotic episodes. Subsequently, we isolated DNA coding for the heavy chain of IgG antibodies from B lymphocytes and via phage-display we selected single chain Fv’s for recognition of β_2_GPI. Both antibodies were reconstructed as complete IgG and displayed LAC activity. Binding characterization using β_2_GPI domain-deleted mutants revealed that both antibodies only react with domain I of β_2_GPI [15].

### Specificity Testing

Reactivity of both antibodies towards epitope G40-R43 on domain I of β2GPI was tested. Therefore we used full-length plasma-purified β_2_GPI [7] and a mutant kindly provided by Dr. Iverson from La Jolla Pharmaceutical Company. In this full-length β_2_GPI mutant, arginine 43 was replaced by a glycine (R43G) [4]. The full-length β2GPI and its mutant R43G (0,2 µM) were coated onto an anionic and/or neutral-charged ELISA 96-wells plate (both Costar, New York, USA). Subsequently P1-117 and P2-6 were added to the coated ELISA plates at different concentrations, ranging from 0 to 25 µg/ml. Bound antibodies were detected by a HRP-labeled goat-anti-human IgG antibody and staining was performed using tetramethylbenzidine (Kordia, Leiden, The Netherlands). Results are expressed as percentages, normalized to the highest concentration of P2-6 (100%).

### Assays

Five ELISA-based anti-β_2_GPI assays were selected based on their availability, and consisted of the REAADS IgG anti- β_2_GPI semi-quantative test kit (Corgenix, Colorado, USA); the Asserachrom® anti- β_2_GPI IgG test (Diagnostica Stago S.A.S., Asnières sur Seine, France); the Varelisa β_2_GPI IgG antibodies test (Phadia GmbH, Freiburg, Germany); the anti-β_2_GPI IgG immunoassay (Orgentec Diagnostica GmbH, Mainz, Germany); the anti-β_2_GPI IgG test (Bio-Rad Laboratories, California, USA). In the result section the assays are encoded. We have decided not to disclose which result correlates to which assay as courtesy to the companies. When manufacturers are interested in how their assay performs, we will provide them with their test results. All tests were performed according to the instructions of manufacturers. Cut-offs from the different assays were used as stated in the disclosure of the kits. Both antibodies P1-117 and P2-6 were titrated in dilution buffer supplied by the manufacturer, and added to the plate at concentrations ranging from 0·001 to 10 µg/ml. OD values were registered and subsequently expressed as percentages, normalized to the highest concentration of P2-6 (100%).

### Patients

This study was conducted according to the revised Helsinki Protocol. All participants gave written informed consent. A first cohort consisted of twenty patients, selected from a large cohort previously diagnosed at the thrombosis center at the University of Padua (Italy) [3]. Approval was obtained by the ‘Comitato Etico dell’Azienda Ospedaliera Universitaria di Padova’ (IRB of Padua University Hospital, Italy). The identification of samples containing antibodies specifically against epitope G40-R43 on domain I of β_2_GPI was described earlier [3], [6]. In total, 10 patients were selected for their reactivity towards epitope G40-R43, varying from low to highly positive, together with 10 negative control patients. A second cohort diagnosed at the University of Lorraine consisted of 172 patients, possibly suffering from APS (including 51 SLE patients). Approval for this cohort was obtained by the ‘Comité Consultatif de Protection des Personnes dans la Recherche Biomédicale de Lorraine’ (France). Samples were tested for their reactivity against domain I of β_2_GPI by ELISA, as described previously [6], and for positivity in assay A and B.

## Results

**Figure 1:**
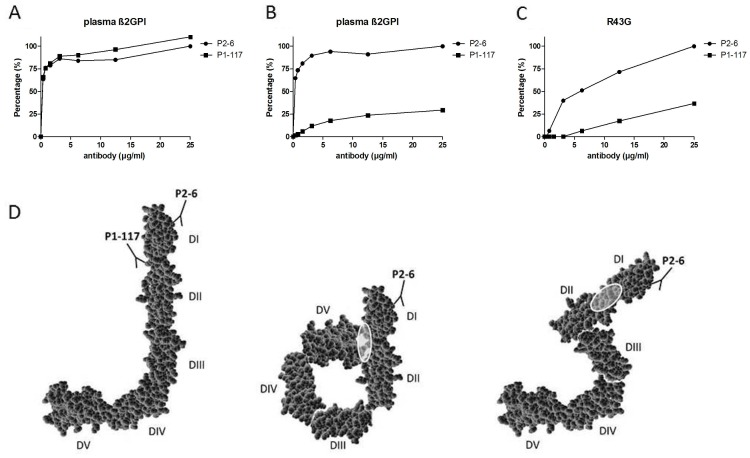
Specificity of monoclonal antibodies P1-117 and P2-6. Two different monoclonal human-derived antibodies to domain I of β_2_GPI, P1-117 and P2-6, were tested for their specificity in an ELISA as described in Material and Methods. Different concentrations (0 to 25 µg/ml) of these antibodies were added to an ELISA plate coated with full-length plasma purified β_2_GPI (A and B) or a full-length β_2_GPI mutant in which arginine 43 was replaced by a glycine (C). These proteins were coated on an anionic plate (A+C) or a neutral plate (B). Results are expressed as the percentage of the maximum OD value. D. β_2_GPI exists in different conformations, an open J-shaped conformation, a circular conformation and a S-shape conformation. The binding sites of P1-117 and P2-6 are indicated. In the circular conformation, interaction of domain I with domain V prevents exposure of the P1-117 epitope. Similarly, in the S-shaped conformation, the carbohydrate residues are positioned in such a way that they shield the P1-117 epitope.

We first investigated the specificity of the human-derived monoclonal anti-domain I antibodies P2-6 and P1-117 [15]. β_2_GPI exists in different conformations, a native conformation and an “activated open” conformation. The latter is induced after interaction with negatively charged surfaces such as phospholipids or LPS [7], [8], [16]. To study the binding of the isolated anti-domain I antibodies towards the native and open conformation, we tested their reactivity to β_2_GPI coated on an anionic versus a neutral ELISA plate. Both antibodies displayed comparable affinity towards plasma-purified β_2_GPI coated on an anionic ELISA plate (Fig. 1 A). Interestingly, antibody P1-117 but not P2-6 almost completely lost its reactivity towards β_2_GPI coated on a neutral-charged ELISA plate. (Fig. 1B). Subsequently, we investigated the reactivity of both antibodies towards a full-length mutant of β_2_GPI in which amino acid 43 (arginine) was replaced by a glycine (R43G), coated on a negatively-charged ELISA plate. P1-117 was found to show a significantly decreased affinity towards this mutant R43G compared to P2-6 (Fig. 1 C). Taken together, these data demonstrate that P1-117 recognizes epitope G40-R43 on domain I of β_2_GPI, exposed only when β_2_GPI is bound to anionic surface, while P2-6 reacts with domain I of β_2_GPI regardless of its conformation.

**Figure 2:**
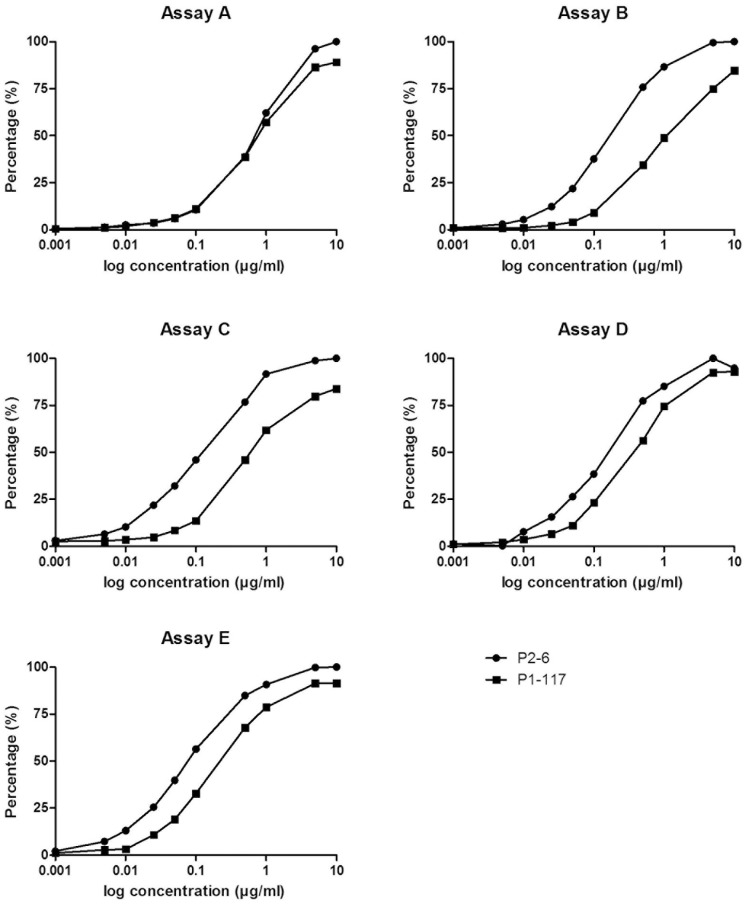
Comparison of the exposure of a domain I epitope G40-R43 of β_2_GPI in commercial anti- β_2_GPI IgG assays. Increasing concentrations, ranging from 0.001 to 10 µg/ml, from the monoclonal antibodies P1-117 and P2-6 are tested in 5 different anti-β_2_GPI assays, as described in Materials and Methods. Results are expressed as the percentage of the maximum OD value of P2-6. Based on the difference in reactivity of P2-6 and P1-117, assays can be evaluated. Results are representative for one out of 2 experiments.

Given the characteristics described above, the antibodies P1-117 and P2-6 can be used to compare the exposure of epitope G40-R43 on β_2_GPI in different commercially available anti-β_2_GPI assays. Therefore, we tested the reactivity of both antibodies in five commercially available anti-β_2_GPI IgG ELISAs, as described in materials and methods. In assay A, P1-117 and P2-6 showed comparable reactivity towards the coated β_2_GPI, indicating all of the coated β_2_GPI is present in its open conformation, exposing epitope G40-R43 (Fig. 2). However, in the other four assays, a significantly lower reactivity of P1-117 to the coated β_2_GPI was found compared to P2-6, suggesting a reduced exposure of epitope G40-R43.

**Figure 3:**
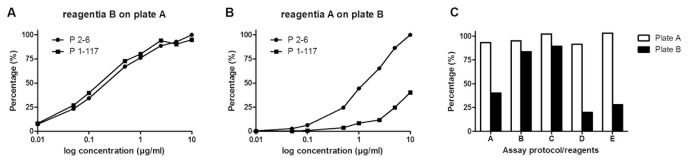
Influence of different assay reagents/protocols on the reactivity of antibodies P1-117 and P2-6 on coated plates of 2 commercial anti-β_2_GPI IgG assays. The reactivity of antibodies P1-117 and P2-6 was tested as explained in Fig. 2 on coated ELISA plates of assay A and B, applying the reagents/protocol of each of the 5 separate assays. Results are expressed as the percentage of the maximum OD value of P2-6. A–B. Graphic representation of results obtained when the protocol and reagents of assay B were applied on a coated plate of assay A (A) and vice versa (B). C. Table showing the reactivity of 10 µg of antibody P1-117 on coated plates of assay A and B using the protocol and reagents of assay A–E.

As the reduced reactivity of antibody P1-117 in assays B–E may also result (in part) from differences in assay features other than the plates used for coating β_2_GPI, we have re-examined the reactivity of antibody P1-117 and P2-6 on coated plates from assay A and B, applying the protocol and reagents from each of the 5 separate assays. Interestingly, we have established comparable reactivity of antibody P1-117 and P2-6 on each coated plate of assay A, regardless of which reagents or protocol were used. For all of the tested reagents/protocols on coated plates of assay B, a reduced reactivity of P1-117 compared to P2-6 was observed. Fig. 3A–B shows the results from the application of the reagents from assay A on a coated plate of assay B and vice versa, while Fig. 3C summarizes the results obtained with 10 µg/ml P1-117 for all the tested combinations. For each of the tested reagents with the corresponding protocol on coated plates of assay A, a reactivity above 90% was established after addition of 10 µg/ml P1-117. For all conditions tested on the plates of assay B, the reactivity of P1-117 was found to be reduced. The huge variability in this reduced reactivity between the different reagents suggests that the coating in plate B is less stable compared to the coating in plate A. However, given the constantly high versus reduced reactivity of P1-117 on plates of assay A versus B, our data suggest that differences in the influence of the plates on the exposure of β_2_GPI epitopes, rather than any other component of the assays, provide an explanation for the observed reduction in reactivity of antibody P1-117 in assays B–E.

Subsequently we verified whether this difference in exposure of epitope G40-R43 might be responsible for the high variability in sample classification between assays of different manufacturers. As a proof of principle, a small number of patient samples were selected from a large cohort described before [3]. Samples were tested for the presence of domain I IgG antibodies previously by an in-house ELISA and based on these results we selected 10 patients reactive to epitope G40-R43 on domain I, together with 10 anti-domain I negative patients. Clinical and serological characteristics of the patients are shown in table 1. Both patient groups consisted mainly of females and the mean age was 36 years. All patients belonging to group 1 had a history of thrombosis (arterial, 60%; venous, 60%), versus only 20% of the patients in group 2 (arterial, 20%). We tested these samples in assay A and B for reactivity against the coated β_2_GPI, as described in Materials and Methods. A perfect correlation was found between domain I reactivity and positivity in assay A (table 2). However, only 3 out of the 10 samples containing anti-domain I antibodies were found to be positive in assay B, the assay in which the availability of epitope G40-R43 was the lowest. Especially the weak positive samples in assay A were found to be negative in assay B.

**Table 1:** Clinical and serological characteristics of the first patient cohort.

	Domain I negative patients (n = 10)	Domain I positive patients (n = 10)
Females, n	8	9
Mean age, y	36	36
Pregnancy morbidity, n	1	2
SLE, n	10	5
LLD, n	0	2
Primary APS, n	0	3
Lupus anticoagulant	2	10
**Clinical symptoms**		
Thrombosis, n	2	10
Arterial thrombosis, n	2	6
Venous thrombosis, n	0	6
**Antibody distribution**		
Anti-B2GPI antibodies, n	1	10
IgG, n	0	10
IgM, n	1	6
Antiprothrombin antibodies, n	5	8
aCL antibodies, n	5	10
IgG, n	4	10
IgM, n	3	7

**Table 2:** Reactivity of patient samples of cohort 1 in two different anti-β_2_GPI assays.

Patient	Thrombosis^1^	APTT/dRVVt^2^	Domain 1 Positivity^3^	Assay A	Assay B
2	Yes	Prolonged	Yes	**+**	**–**
3	Yes	Prolonged	Yes	**+**	**–**
4	Yes	Prolonged	Yes	**+**	**–**
5	Yes	Prolonged	Yes	**++**	**–**
6	Yes	Prolonged	Yes	**+++**	**+++**
9	Yes	Prolonged	Yes	**++**	**–**
10	yes	Normal	Yes	**+++**	**+++**
13	Yes	Prolonged	Yes	**+**	**–**
17	Yes	Prolonged	Yes	**+**	**–**
19	Yes	Prolonged	Yes	**++**	**++**
1	No	Normal	No	**–**	**–**
7	Yes	Prolonged	No	**–**	**–**
8	Yes	Normal	No	**–**	**–**
11	No	Normal	No	**–**	**–**
12	No	Normal	No	**–**	**–**
14	No	Prolonged	No	**–**	**–**
15	No	Normal	No	**–**	**–**
16	No	Normal	No	**–**	**–**
18	No	Normal	No	**–**	**–**
20	No	Normal	No	**–**	**–**

20 selected patient samples, with the indicated characteristics, were tested for reactivity against coated β2GPI in assay A and assay B. Samples were considered strong positive (+++), positive (++) and weak positive (+) according to the recommendations of the manufacturers.

1 Arterial or venous thrombosis.

2 Activated partial thromboplastin time/***dilute Russell's Viper Venom time.***

3 Antibody reactivity against epitope G40-R43 of domain I of β_2_GPI, as described previously [3], [4], [5].

Subsequently, a large cohort containing 172 samples from patients with suspected APS, including 51 SLE patients, was tested for reactivity against domain I of β_2_GPI by ELISA, as described previously [6]. 23 out of 172 samples were found to be positive for reactivity against domain I. Clinical and serological characteristics of domain I negative and reactive samples are shown in table 3. All samples were applied on assay A and B, and results are shown in table 4. 22 out of 23 domain I-positive samples tested positive in assay A, versus 19 in assay B. From the 149 domain I negative samples, 11 and 8 samples were shown to be reactive against the coated β_2_GPI of assay A and B, respectively (data not shown). These domain I-negative samples may be reactive against other domains of β_2_GPI, rendering it difficult to identify potential false-positive results. Importantly, we did confirm in this second cohort that a number of domain I-reactive samples can be missed in assays characterized by a decreased exposure of epitope G40-R43.

**Table 3:** Clinical and serological characteristics of cohort 2 (172 patients).

	Domain I negative patients (n = 149)	Domain I positive patients (n = 23)
Females, n	109	19
Mean age, y	43	45
Pregnancy morbidity, n	14	4
SLE, n	44	7
LLD, n	0	0
Primary APS, n	67	9
Lupus anticoagulant	69	22
**Clinical symptoms**		
Thrombosis, n	77	10
Arterial thrombosis, n	27	4
Venous thrombosis, n	45	7
Small vessel thrombosis, n	8	2
**Antibody distribution**		
Anti-B2GPI antibodies, n	35	22
IgG, n	9	19
IgM, n	28	13
Antiprothrombin antibodies, n	81	20
aCL antibodies, n	50	22
IgG, n	26	20
IgM, n	29	12

**Table 4:** Reactivity of SLE/APS patient samples from cohort 2 in two different anti-β_2_GPI assays.

Patient	Domain 1 Positivity^4^	Assay A	Assay B
1	Yes	**+++**	**+++**
2	Yes	**+++**	**+++**
3	Yes	**+**	**–**
4	Yes	**+**	**+**
5	Yes	**++**	**+++**
6	Yes	**++**	**+++**
7	Yes	**++**	**++**
8	Yes	**++**	**+++**
9	Yes	**++**	**++**
10	Yes	**+++**	**+++**
11	Yes	**+**	**–**
12	Yes	**+++**	**+++**
13	Yes	**+++**	**+++**
14	Yes	**+++**	**+++**
15	Yes	**–**	**–**
16	Yes	**+++**	**+++**
17	Yes	**+++**	**+++**
18	Yes	**++**	**+**
19	Yes	**++**	**+++**
20	Yes	**++**	**+++**
21	Yes	**++**	**++**
22	Yes	**+++**	**+**
23	Yes	**+**	**–**

172 patient samples with suspected APS (including 51 SLE patients) were tested for reactivity against coated β_2_GPI in assay A and assay B. Results are shown for the 23 domain I-reactive samples. Samples were considered strong positive (+++), positive (++) and weak positive (+) according to the recommendations of the manufacturers.

1 Antibody reactivity against domain I of β_2_GPI, as described previously [6].

## Discussion

Many antigens have been found to be associated with APS, but β_2_GPI, and more specifically domain I of β_2_GPI, is regarded as clinically most relevant. Indeed, patients with antibodies specifically against epitope G40-R43 on domain I have a 18.9 fold increased risk of thrombosis compared with patients with antibodies to other domains [6]. Correspondingly, Ioannou *et al.* have demonstrated that the pathogenic epitope in domain I comprises the G40-R43 region together with the interlinker region between domain I and II [5]. So far, no pathogenic antibodies have been identified against other parts of domain I. β_2_GPI has been described to adopt different conformations. In 1999 the crystal structure of β_2_GPI revealed a J-shaped conformation [10], [11]. In addition, by applying small-angle X-ray scattering, Hammel *et al.* showed in 2002 that β_2_GPI in solution is present in an S-shape [9]. In this conformation, epitope G40-R43 on domain I is blocked by the presence of a carbohydrate chain [7], [9]. Recently Agar *et al*. have demonstrated that native β_2_GPI adapts to a circular conformation [8]. We have published that epitope G40-R43 is cryptic and hidden in native β_2_GPI [7]. However, upon interaction with anionic surfaces such as phospholipids, β_2_GPI changes its conformation (either S-shaped or circular into J-shape), thereby exposing epitope G40-R43 and enabling antibodies to bind. Recently, we have cloned anti-domain I antibodies from two patients using phage display [15]. Using different ELISA plates, we have now provided evidence that antibody P1-117 recognizes epitope G40-R43, exposed only when β_2_GPI is in the open conformation, as induced by interaction with anionic surfaces. In contrast, antibody P2-6 was able to interact with domain I of β_2_GPI regardless of its conformation and independent from the exposure of epitope G40-R43. Importantly, these two antibodies can be used to distinguish between the different native and open conformations of β_2_GPI, characterized by the block or exposure of epitope G40-R43, respectively.

Antibodies correlating with an increased risk for thrombosis have been shown to especially recognize this epitope G40-R43 of domain I of β_2_GPI [3], [4], [5]. In the next set of experiments the isolated P1-117 and P2-6 antibodies were used to measure the exposure of this epitope G40-R43 on domain I in anti-β_2_GPI IgG assays of different manufacturers. Interestingly, we observed two patterns of reactivity of antibodies P2-6 and P1-117. In one assay both antibodies showed comparable reactivity towards the coated β_2_GPI, indicating most of the coated β_2_GPI is present in the J-shaped conformation exposing epitope G40-R43 (figure 2, assay A). The second pattern was characterized by a decreased affinity of P1-117 towards the coated β_2_GPI compared to antibody P2-6 (figure 2, assay B,C,D,E). In these assays, a significant part of β_2_GPI is present in its native conformation, resulting in a decreased availability of epitope G40-R43. Interestingly, the difference in reactivity between P1-117 and P2-6 in each individual assay appeared to be different and probably correlates with the amount of β_2_GPI exposing epitope G40-R43. By testing the reagents/protocol of each of the 5 assays on the coated plates of assay A and B, we have demonstrated that indeed a difference in the ELISA plates used, rather than any other component of the assays, is responsible for the difference in reactivity of both antibodies. Taken together, our results clearly indicate differences in exposure/availability of epitope G40-R43 on domain I of β_2_GPI between commercially available assays.

A major problem within APS is the variability between different commercial anti-β_2_GPI available assays [2], [12], [13], [14]. External quality Control of diagnostic ***Assays*** and Tests Disagreement (ECAT) reports showed that laboratories obtain good results for negative or clearly positive samples. However, there is still a wide variability in sample classification when the sample is a weak positive. Various studies tried to find the origin of this high variability, in the different ways to calculate cut-offs (using the 99^th^ percentile instead of the mean and standard deviation), lack of reference calibrators, different plates used for the coating, different sources of the antigen, use of different buffers, use of mono- versus polyclonal antibodies,… [17], [18], [19], [20], [21]. However, so far no clear-cut explanation for this high variability is found. To verify if this variability may arise from the differences in exposure of epitope G40-R43, we tested samples from patients which were tested earlier for the presence or absence of anti-β_2_GPI domain I antibodies using an in-house ELISA. In assay A, all 10 patients with anti-G40-R43 antibodies tested positive. However only 3 out of 10 positive patients tested positive in assay B. Similarly, in a second cohort of 172 consecutive patients with suspected APS, 22 out of 23 domain I-positive samples were found to be positive in assay A, compared to 19 samples in assay B. From the 149 domain I negative samples, 11 versus 8 samples were shown to be reactive against the coated β_2_GPI of assay A and B, respectively. Probably, these samples contain antibodies reactive against other domains of β_2_GPI, for which a correlation with thrombosis has not been demonstrated yet. Although it is impossible to evaluate the test results from the domain I negative samples, we have provided clear evidence for an increased risk to falsely assign domain I-reactive samples as being negative in assays characterized by a decreased exposure of epitope G40-R43. Noteworthy, adaptation of the cut-off value from assay A/B did not ameliorate test results, indicating that the difference in patient classification is not simply a result from different cut-off settings (data not shown).

Our results clearly indicate that a significant number of APS patients can be missed in assays that do not expose epitope G40-R43 properly, resulting in underdiagnosis of the syndrome. These false-negative results have serious consequences for patients suspected of having APS since they need long-term anticoagulation to prevent recurrence. Despite the high diversity of reagents and the heterogeneity in methodology between the two assays, we have clear indications that the observed discrepancies in reactivity arise from differences in antigen coating, more precisely the exposure of the cryptic epitope G40-R43. We are not suggesting that in some of the commercially available assays β_2_GPI does not expose G40-R43 at all, but have provided evidence that a significant part of the coated β_2_GPI in some assays is present in a conformation that does not allow the binding of all domain I auto-antibodies. This reduced exposure seems to result in lower sensitivity for low titer antibodies. Importantly, as a result some domain I-positive samples, especially the weak positive ones, can be missed in such assays. Noteworthy, in an assay such as assay A, in which all the coated β_2_GPI exposes the cryptic epitope G40-R43, all other domains probably remain accessible for binding. This is important given the fact that so far insufficient evidence exists to claim that only domain I contains pathogenic epitopes. If further research accomplishes that only domain I harbors epitopes for antibodies correlating with an increased risk for thrombosis, assays coating only domain I of β_2_GPI will be interesting since they are less sensitive to antigen preparation and coating, as they do not require a conformational change. If further research identifies pathogenic epitopes in other domains, existing assays need to be improved to ensure that at least the epitopes which have shown to be pathogenic are exposed correctly. Also, awaiting the multicenter studies comparing the sensitivity of assays coating only domain I versus the whole β_2_GPI, we need to rely on the existing assays, supporting the need to urgently understand the variability between the assays and optimize them to prevent misdiagnosis. Of note, studies have shown that in the new generation assays the measurement of anti-cardiolipin and anti-β_2_GPI antibodies correlate well. These results were to be expected as the anti-cardiolipin assays use β_2_GPI as a coating/reagent. The conformation of β_2_GPI may therefore influence the anti-cardiolipin detection as well. In an anti-cardiolipin and anti-β_2_GPI assay produced by the same manufacturer, the conformation of β_2_GPI will probably be the same. Interestingly, this may explain why not all APS patients are triple positive and a large part even have isolated LA positivity.

In conclusion, by using two monoclonal antibodies with different reactivity towards β_2_GPI, we have demonstrated clear differences in exposure of epitope G40-R43 on domain I of β_2_GPI in commercial anti-β_2_GPI IgG assays. Furthermore, we found that a significant number of samples positive for domain I reactivity are falsely assigned negative in assays characterized by a decreased availability of this epitope. One way to avoid the variability resulting from differences in antigen presentation in the commercial anti-β_2_GPI assays, would be to use negatively-charged ELISA plates, mimicking the physiologic binding of β_2_GPI to phospholipids, and resulting in complete exposure of the epitope G40-R43 correlating with an increased risk for thrombosis. We further propose to use monoclonal antibodies similar to P1-117 and P2-6 as controls in any commercial assay used to diagnose APS to ensure satisfactory exposure of the pathogenic epitope G40-R43. This will ascertain us that, together with the other β_2_GPI-reactive patients, also the ones reactive against the pathogenic G40-R43 epitope are not falsely assigned negative. In the meantime, studies investigating other domains for the presence of pathogenic epitopes and determining the sensitivity of assays coating only domain I are urgently needed.
